# Primary renal adenosquamous carcinoma

**DOI:** 10.4103/0974-7796.68862

**Published:** 2010

**Authors:** Mohammad Ashik Zainuddin, Tan Yeh Hong

**Affiliations:** Department of Orthopaedic Surgery, Singapore General Hospital, Singapore; 1 Department of Urology, Singapore General Hospital, Singapore

**Keywords:** Adenosquamous carcinoma, metaplasia, renal

## Abstract

A case of renal adenosquamous carcinoma is presented. The fact that the urothelium has no glandular or squamous structures makes the pathogenesis of this tumor unique. The process is assumed to begin with urothelial metaplasia resulting from chronic irritation leading to dysplasia and subsequently squamous and glandular differentiation.

## INTRODUCTION

Primary adenosquamous carcinoma of the kidney is a rare phenomenon. We describe a case of a 62-year-old diabetic male with previous bilateral staghorn calculi who presented to our unit with anemia, painless left hypochondrial mass, microhematuria and leukocytosis.

## CASE REPORT

A 62-year-old male, with a past history of diabetes mellitus, hypertension and chronic renal impairment had bilateral staghorn calculi for which he underwent bilateral percutaneous nephrolithotripsy (PCNL) 6 years ago. He also underwent left kidney double-J stenting 1 year ago for left hydronephrosis.

He was initially admitted to the gastroenterology unit for diarrhoea of 2 weeks duration. There was no fever, loss of weight or loss of appetite. On clinical examination, he was afebrile but was noted to have mild pallor. Abdominal examination revealed a nontender left hypochondrial mass. Per rectal examination was normal.

Blood investigations showed leukocytosis with raised total white blood cells of 31×10^9^ units/l. The hemoglobin level was 9.7 g/dl with a microcytic hypochromic picture. Septic workup which included blood cultures, urine culture and a chest radiograph was normal. Urine microscopy showed microhematuria and pyuria.

**Figure 1:**
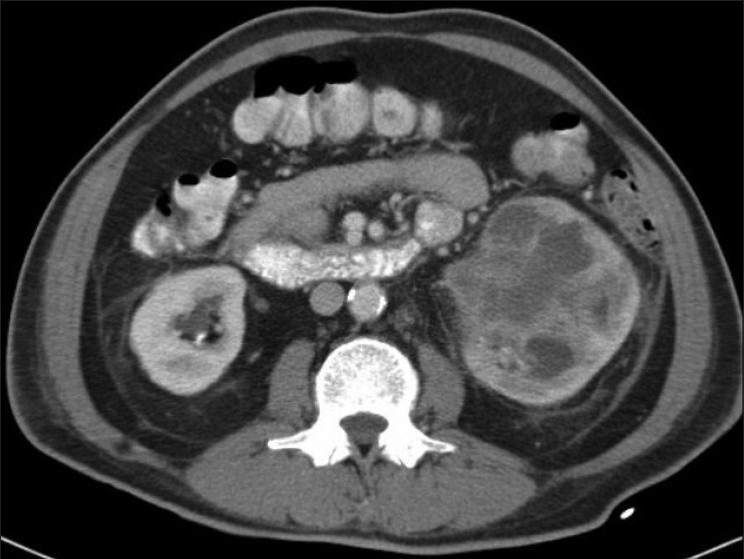
CT scan of the kidneys demonstrating large lower pole heterogenous mass with perirenal fat stranding

Gastroscopy and colonoscopy demonstrated antral gastritis and ascending colon diverticula, respectively. An ultrasound of the abdomen revealed a left renomegaly secondary to a large complex septated cyst with internal debris and left renal lower pole calculi. A computed tomographic (CT) scan of the kidneys demonstrated a large 7.8-cm heterogenous lesion in the lower pole of the left kidney with perirenal fat stranding, and adjacent enlarged para-aortic lymph nodes [Figure 1].

The patient underwent an open left radical nephrectomy, and intraoperatively was found to have large left renal lower pole tumor adherent to, but not involving, the posterior peritoneum with edematous perirenal fat.

Histologic finding was that of a poorly differentiated adenosquamous carcinoma involving the renal pelvis. There was perineural and perirenal fat invasion. The remaining specimen showed hydronephrosis, chronic pyelonephritis and focal atypical squamous metaplasia of the pelvis. Surgical margins were cleared of tumor.

In the postoperative period, the patient developed enterococcus wound infection which resolved with intravenous antibiotics. He was discharged well and was subsequently referred to the oncologist for chemotherapy.

**Figure 2:**
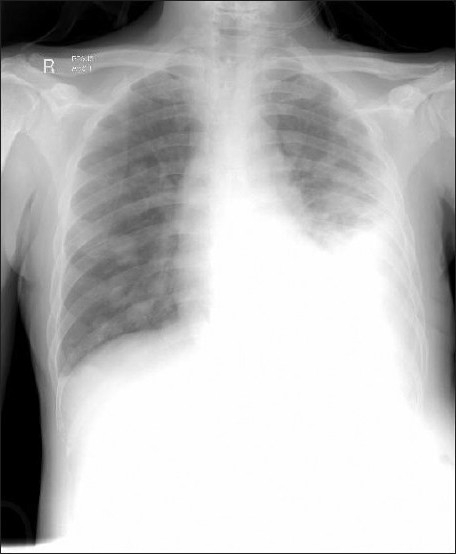
Chest radiograph taken 3 months postoperatively showing lung metastasis and a moderate left pleural effusion. Preoperative chest radiograph was normal

Three months after surgery, the patient was admitted for complains of shortness of breath. A chest radiograph done showed multiple cannon ball lung lesions and a moderately sized left pleural effusion [Figure 2] The effusion was drained and he was given palliative chemotherapy. He succumbed to his illness 6 months postsurgery.

## DISCUSSION

Primary adenosquamous carcinoma of the kidney is rare with only a handful of cases reported in the medical literature.[1,2] Secondary adenosquamous carcinoma can arise in the kidneys from various sources, mainly the lungs[3] and pancreas.

Primary adenosquamous tumors are associated with calculi, hydronephrosis and pyelonephritis. Hence, chronic irritation due to recurrent infections is assumed to be an etiologic factor.[4–6] Chronic irritation by renal calculi and infection causes squamous and glandular metaplasia with subsequent adenosquamous neoplasia.[1,7]

**Figure 3:**
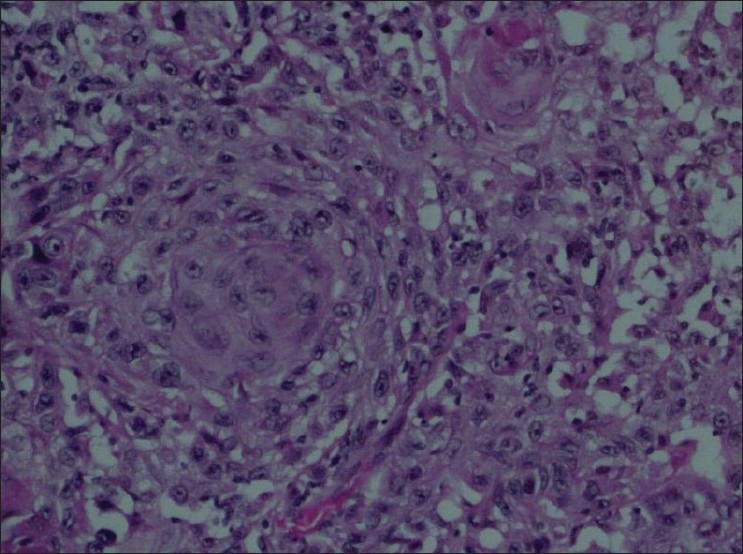
Microscopic view of tumor specimen demonstrating pleiomorphism and two keratin whorls, suggestive of differentiation towards a squamous cell carcinoma (H and E stain, ×100)

**Figure 4:**
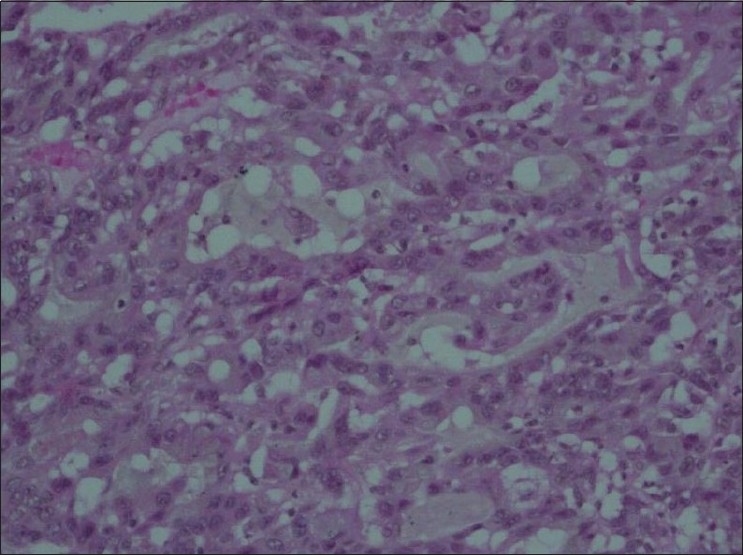
Microscopic view of the same tumor specimen showing malignant cells adopting a glandular architecture.(H and E stain, ×100)

Key histologic features noted in this case were that of sheets of pleiomorphic cells with foci of squamous differentiation (keratinized squamous pearls) [Figure 3] and glandular differentiation (glands containing intraluminal mucin) [Figure 4].

Renal adenosquamous carcinoma follows an aggressive course. It can metastasize to locoregional areas[8] or to distal sites via hematogenous route. As can be seen from this case, the patient had become symptomatic from metastatic disease within 3 months of diagnosis of the disease. The most important prognostic factor is still the pathologic stage.

We learnt that from this case that although primary renal adenosquamous carcinoma of the renal pelvis is a rare entity, it should be considered as one of the differential diagnosis in the evaluation of patients with microhematuria. This is because clinical presentation is atypical, and hence, could lead to a delay in diagnosis and treatment of this aggressive disease.
